# A Longitudinal Case-Control Study of a Female Athlete Preinjury and After ACL Reconstruction: Hop Performance, Knee Muscle Strength, and Knee Landing Mechanics

**DOI:** 10.1177/19417381221147305

**Published:** 2023-02-06

**Authors:** Josefine E. Naili, Jonas L. Markström, Charlotte K. Häger

**Affiliations:** † Department of Women’s and Children’s Health, Karolinska Institutet, Stockholm, Sweden; ‡ Department of Community Medicine and Rehabilitation, Unit of Physiotherapy, Umeå University, Umeå, Sweden; § Umeå School of Business, Economics and Statistics, Unit of Statistics, Umeå University, Umeå, Sweden

**Keywords:** 3-D motion analysis, hop testing, strength testing

## Abstract

Athletes with an anterior cruciate ligament (ACL) injury followed by ACL reconstruction (ACLR) often perform various testing to guide return to sport, but preinjury data are rarely available for comparison. This longitudinal case-control study reports absolute value and between-leg symmetry data on maximal performances for single-leg hop height and distance, muscle strength, and side hop landing mechanics of an 18-year-old female soccer athlete collected 5 months before sustaining an ACL injury and again at 10, 13, and 29 months post-ACLR. Her data were compared across test sessions and to cross-sectional data of 15 asymptomatic female athletes.

Few studies report anterior cruciate ligament (ACL) preinjury and repeated post-ACL reconstruction (ACLR) testing for functional performances and biomechanics. Case reports have presented pre- and post-ACL injury data for knee strength and gait mechanics,^5^ and performances and joint mechanics during the star excursion test, hop for distance, and sidestep cutting.^6-8^ These results suggest that regaining preinjury results differ between functional performances and knee biomechanics. However, further pre- and post-ACL injury comparisons are needed to better grasp the consequences of injury.

We had the opportunity to compare longitudinally data on hop performances, muscle strength, and knee mechanics of an 18-year-old female elite athlete collected 5 months before she suffered an ACL injury with data collected again at 10, 13, and 29 months post-ACLR.

## Case Report

The injured athlete (IA; body height, 1.64 m; body mass index, 21.3 kg/m^2^) played soccer in the second-highest national division and sustained a contact ACL injury on her nondominant (nonpreferred leg to kick a ball) leg. The injury was verified using magnetic resonance imaging and treated with ACLR using a hamstring tendon autograft 1 month after injury. The IA completed 13 months of physiotherapist-led rehabilitation (Appendix Table A1, available in the online version of this article). Before fully returning to soccer, standardized evaluation was performed at the outpatient physiotherapy clinic attended by the IA at 10 and 13 months postsurgery (Appendix Table A2, available online). She had no history of injury or surgery to the back or lower extremities. The data from the IA were compared across test sessions and to cross-sectional data of 15 asymptomatic female athletes (mean [SD]: age, 21.6 [2.6] years; body mass index, 22.4 [2.2] kg/m^2^) competing in the highest/second-highest national divisions in floorball or soccer. These athletes were screened for musculoskeletal or neurological pathology before testing.

Participants performed, barefoot, and in the following order, the maximal tests 1-leg hop for distance (OLHD, aiming for maximal distance and landing on the same leg while maintaining balance) and 1-leg vertical hop height (OLVH, hopping upward from standing on 1 leg, aiming for maximal height and landing on the same leg), the standardized rebound side hop (SRSH, hopping on 1 leg laterally to the side over a distance of 25% of body height and immediately rebounding back to the starting position for the same leg) for biomechanical evaluation, and isometric peak knee extension and flexion strength (knee in ~65° flexion) using a KinCom isokinetic dynamometer. Limb symmetry indexes (LSIs, injured leg/noninjured leg × 100) were calculated for peak values for OLHD, OLVH, and strength tests. Jump distance was also reported in percentage of body height. The biomechanical analysis of knee joint mechanics in frontal and sagittal planes was performed using a model of 56 passive spherical markers and an 8-camera Qualisys motion capture system (240 Hz) synchronized with 2 Kistler force plates (1200 Hz).^3^ Data from the IA outside the 95% CI of the mean value data among controls were interpreted as atypical.

## Results

**Figure 1.**
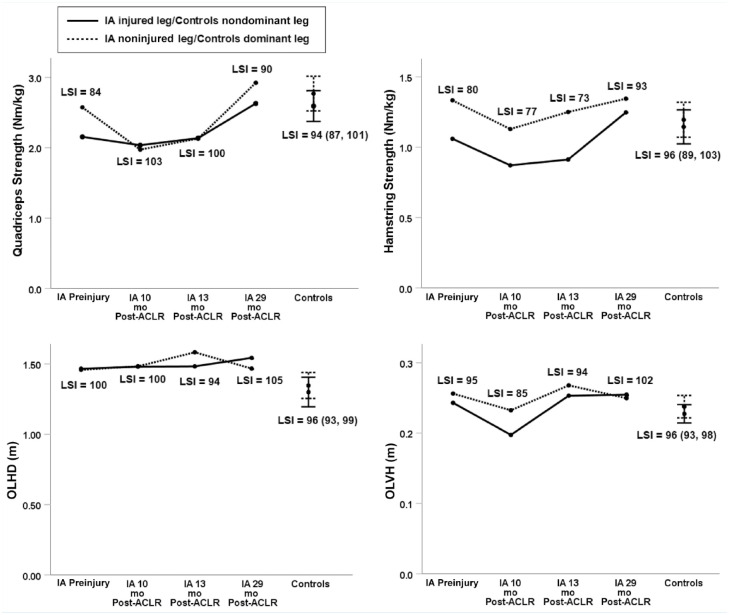
LSIs for knee extension and flexion strength, OLHD and OLVH for the IA before and after ACLR, and 15 asymptomatic athletes. ACL, anterior cruciate ligament; ACLR, ACL reconstruction; IA, injured athlete; LSI, limb symmetry index; OLHD, 1-leg hop for distance; OLVH, 1-leg vertical hop height.

**Figure 2.**
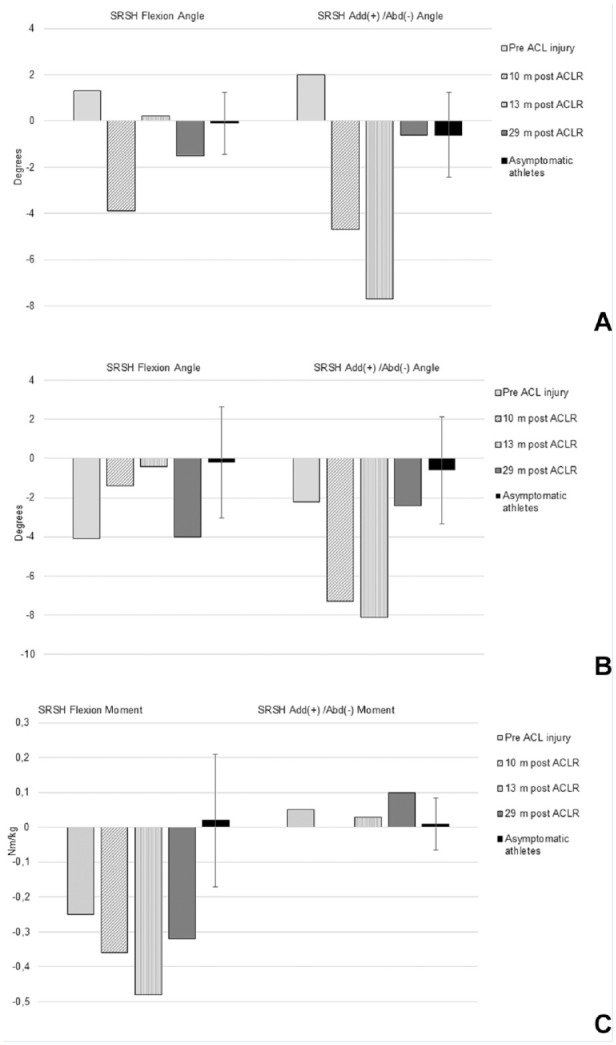
(A) Knee flexion and add/abd angles at initial contact. (B, C) knee flexion and add/abd angles (B) and moments (C) during the landing phase. Injured minus noninjured leg for the IA; nondominant minus dominant leg with 95% CI for asymptomatic athletes. abd, abduction; ACL, anterior cruciate ligament; ACLR, ACL reconstruction; add, adduction; IA, injured athlete; m, months; SRSH, standardized rebound side hop.

The IA regained preinjury OLHD and knee extension strength at 10 months post-ACLR, OLVH at 13 months post-ACLR, and knee flexion strength at 29 months post-ACLR. Peak OLHD in percentage of body height was, at preinjury testing, 89%/89% (soon-to-be injured/noninjured), at 10 months post-ACLR 90%/90% (injured/noninjured), at 13 months post-ACLR 90%/97%, and at 29 months post-ACLR 95%/89%, whereas controls on average demonstrated 79% (SD 12%)/82% (SD 10%) (nondominant/dominant leg). The knee extension and flexion strength of the IA for the noninjured leg decreased after injury and was not regained until the 29-month test (Figure 1). During SRSH, she displayed lower knee flexion angles (~13-17° less) but higher knee flexion moments (~0.3-0.6 Nm/kg more) and more asymmetry for knee flexion moments (~0.3-0.5 Nm/kg leg-difference) and adduction-abduction angles (~5-8° leg-difference) post-ACLR compared with preinjury (Figure 2). The knee flexion moment was similar to controls during preinjury testing but was higher for all post-ACLR tests (~0.3-0.5 Nm/kg more).

## Discussion

Our main finding was that the recovery profile of hop performances, knee strength, and knee landing mechanics of the IA was neither congruent nor fully regained upon returning to competitive sports. The IA completed 13 months of physiotherapist-led rehabilitation while progressively participating in sport-specific training with her soccer team, decided in agreement with the team professionals. Knee flexion strength took the longest to regain, likely explained by the hamstring graft donor site. Preinjury knee extension strength of both limbs was lower than for the controls and never reached the commonly referred to clinical norm of 3.0 Nm/kg despite increasing over the course of, and beyond, rehabilitation. These results corroborate previous findings showing that recovery differs across outcome measures from preinjury to post-ACLR.^5-8^

The often-used LSI criteria have been criticized because they do not relate to normative data or account for the decreased function of the noninjured leg and, therefore, overestimate the ACLR leg.^9^ We observed this effect primarily for knee extension strength, where the asymmetric preinjury LSI of the IA was symmetric at 10 and 13 months post-ACLR due to reduced strength for the noninjured limb. Similar to the preinjury data of the IA, we recently reported that most athletes, irrespective of having had ACLR or being noninjured, fail 90% LSI criteria in a test battery of hop and strength tests.^2^ These matters are important when considering outcomes and return-to-sport decisions post-ACLR.

The asymmetrical knee landing mechanics of the IA post-ACLR corroborate previously demonstrated asymmetric gait mechanics long after achieving symmetrical strength and high functional performance.^1^ An asymmetric movement pattern may reflect permanent deficits in sensorimotor knee function not restored by ACLR.^4^

In conclusion, after ACLR, the time needed for the IA to recover her preinjury performances and between-leg symmetry in single-leg hops and muscle strength differed. Despite demonstrating symmetric hop and strength performances, she persistently showed asymmetric side hop landing mechanics. These findings highlight the need for multiple outcomes to inform return-to-sports decisions post-ACLR.

## Supplemental Material

sj-docx-1-sph-10.1177_19417381221147305 – Supplemental material for A Longitudinal Case-Control Study of a Female Athlete Preinjury and After ACL Reconstruction: Hop Performance, Knee Muscle Strength, and Knee Landing Mechanics Click here for additional data file. Supplemental material, sj-docx-1-sph-10.1177_19417381221147305 for A Longitudinal Case-Control Study of a Female Athlete Preinjury and After ACL Reconstruction: Hop Performance, Knee Muscle Strength, and Knee Landing Mechanics by Josefine E. Naili, Jonas L. Markström and Charlotte K. Häger in Sports Health: A Multidisciplinary Approach
