# Supplementary material for: A Longitudinal Case-Control Study of a Female Athlete Preinjury and After ACL Reconstruction: Hop Performance, Knee Muscle Strength, and Knee Landing Mechanics
Source: Sports Health. 2023 Feb 6;15(3):357–60. doi: 10.1177/19417381221147305 (PMC10170219; doi:10.1177/19417381221147305)
Supplement: sj-docx-1-sph-10.1177_19417381221147305 – Supplemental material for A Longitudinal Case-Control Study of a Female Athlete Preinjury and After ACL Reconstruction: Hop Performance, Knee Muscle Strength, and Knee Landing Mechanics [file sj-docx-1-sph-10.1177_19417381221147305.docx]

**ONLINE APPENDIX**

**Table A1. Summary of the injured athlete’s postoperative rehabilitation up to one year following Anterior Cruciate Ligament Reconstruction (ACLR).**

| **Time period**  **post ACLR** | **Exercises** | **Dose** | **Aims/goals and additional instructions** |
| --- | --- | --- | --- |
| Week 3-4 | A) Stationary bike  B) Full knee extension, quadriceps focus  C) Squat with BW, heels raised, max 90° knee flexion  D) Isometric knee flexion (in different joint angle positions)  E) Box step-up  F) Knee extension, supine position, focus on eccentric hamstring work  G) Bi-/unilateral heel raises in standing  H) One limb quiet standing with slight knee flexion, flat 🡪 soft surface | 10-30 min/day 10 reps x 3, 2 times/day  20 reps x 2, 2 times/day (C-G)  20 s x 3/leg, 2 times/day | - Improve range of motion, and muscle function - Walk without limp - Squat with equal and full body weight - Control partial range of motion using hamstrings |
| Week 5-7 | A) Stationary bike or rowing machine B) Glute bridge C) Overhead squat D) Seated leg press (max 90° knee flexion)  E) Standing leg raise cable  F) Box step-up and step-down (focus on the eccentric work)  G) Bi-/unilateral heel raises in standing  H) Full knee extension, quadriceps focus (ACLR limb only) | 10-60 min, 5-6 times/week  10-15 reps x 3, 5-6 times/week 12 reps x 3, 3 times/week  15 reps x 3, 3 times/week 10 reps/leg x 3, 3 times/week  8 reps/leg x 3, 3 times/week  20 reps x 2, 3 times/week  10 reps x 3, 3 times/week | - Walk without limp - Daily: Full range of knee extension - Walk backward with full knee extension in the stance phase - Rest in supine with ACLR leg elevated |
| Month 2-4.5 | A) Movement series using barbells (squat to overhead press) B) Unilateral glute bridge, hold 3 sec in the top  C) Forward lunges  D) Seated leg press, bi-/unilateral eccentric phase (max 90° knee flexion)  E) Lying or sitting leg curl  F) Seated unilateral leg extension*  G) Bilateral heel raises in standing with barbells  H) Side plank and side-lying glute activation  I) Single leg deadlift to overhead press with weights | 8 reps (warm-up)  6 reps x 3  12 reps x 3  12 reps x 2 (bi), 12 reps x 3 (uni)  15 reps x 3  12-20 reps x 3/leg  12 reps x 3  6-10 reps/side x 3  6 reps/leg x 3 | - Strength training 2-3 times/week - Cardiovascular training 1-3 times/week in combination with glute exercises - Full rest 1 day/week |
| Month 4.5-5.5 | A) Seated leg press, bi-/unilateral eccentric phase (max 90° knee flexion)  B) Lying or sitting leg curl, bi-/unilateral eccentric phase ACLR limb  C) Front squat with barbells (max 90° knee flexion)  D) Partial range one leg squat  E) Glute bridge with feet placed on a ball  F) Bilateral heel raises in standing with barbells  G) Alternating leg + arm extension (‘Superman’) on ball  H) Single leg deadlift to overhead press with weights | 12 reps x 2 (bi), 12 reps x 3 (uni)  12 reps x 2 (bi), 15 reps x 3 (uni)  10 reps x 3  6 reps/leg x 3  10 reps x 3  12 reps x 3  10 reps x 3  6 reps/leg x 3 | - Strength training 2-3 times/week - Full rest 1 day/week |
| Month 5.5-7 | A) Squat  B) Deadlift  C) Bulgarian split squat  D) Forwards lunges  E) One leg squat in Smith machine  F) Squat in Smith machine  G) Glute bridge with feet placed on ball  H) Romanian deadlift | 6 reps x 4 (A-H) | - Strength training 2 times/week: including 4 exercises, + eccentric exercises from previous programs* - Complement with core strength exercises |

Reps, repetitions; Bi, bilateral; Uni; unilateral.

**Table A1. Continue…**

| **Time period**  **post ACLR** | **Exercises** | **Dose** | **Aims/goals and additional instructions** |
| --- | --- | --- | --- |
| Month 7-8 | A) Treadmill jog 1.5-2 % inclination 🡪 jog on a soft surface (i.e., thick mat) B) Rope jumping  C) Jog + jump on Bosu-ball  D) Side hop Bosu-ball  E) Skate jump  F) Box jump  G) Explosive lunges  H) Step-up with barbells  I) Hip thrusts in Smith machine  J) Glute bridge variations | 2 min intervals, active rest. Increase every other workout  6 reps x 3 (C-H)  6-10 reps x 3  6 reps/leg x 3 | - Strength training 2 times/week - Increase shock loading 2 times/week, 2 min intervals, active rest. Increase every other workout |
| Month 8-12 | A) Jog + jump on Bosu-ball B) Side hop Bosu-ball  C) Lunge jump on Bosu-balls (each foot placed on one ball) D) Explosive box jumps E) Lunge jump on a flat surface F) One leg jump for distance G) Side hop  H) Glute bridge variations (unilateral, + switch stance leg)  I) Bulgarian split squat + jump (with bodyweight) J) Split squats in Smith machine K) Step-ups with barbells L) Hip thrusts in Smith machine  M) Seated leg press, leg curl, leg extension with unilateral concentric phase | 6 reps x 3 (A-G)  4-6 reps x 3-4 (I-K)  6-10 reps x 3  6 reps x 3 (2 set uni with ACLR limb, 1 set with non-injured limb) | - Strength training 2 times/week - Choose 3 jump exercises, 2 one-leg exercises, and 2 hamstrings focused exercises - Cardiovascular training 2-3 times/week (jogging in total 20 min, cycling intervals 3-4 min) |

Reps, repetitions; Bi, bilateral; Uni; unilateral.

**Table A2. Summary of the injured athlete’s standardized post-operative evaluation at her outpatient physiotherapy clinic.**

| **Time-period post ACLR** | **Standardized evaluation** | **Result and comment** |
| --- | --- | --- |
| Month 10 | Isokinetic knee extension and flexion strength, and hop tests | The IA presented generally satisfying results, although weak knee flexion strength.  The IA returned to soccer practice with restricted contact with opponents. |
| Month 13 | Isokinetic knee extension and flexion strength, and hop tests | The IA presented a ~15% difference between legs in knee flexion and extension strength.  At this time, she had returned to playing soccer matches, although not full-time matches. She reported stiffness and swelling of the knee joint at night after high-intense activity. |
